# OP40 Circumstances That Unlock Payer Acceptability Of Innovative Trial Designs: A Case Study And Primary Research-Based Global Analysis

**DOI:** 10.1017/S0266462324001028

**Published:** 2025-01-07

**Authors:** Sudha Sundaram, Vesshnu Sutharsan, Catherine Kielar, Emil Nørtoft

## Abstract

**Introduction:**

Innovative designs have been used to enhance trial efficiency and enable access to novel treatments when traditional randomized controlled trials (RCTs) were considered operationally challenging to implement. Besides demonstrating scientific and regulatory rigor, innovative approaches also need to be perceived favorably by payers. This work delves into payer perspectives on innovative designs, identifying circumstances that contribute to higher payer acceptability.

**Methods:**

Using targeted searches, we mapped clinical trials since 2010 with novel design elements, including adaptive, master protocol, hybrid, enrichment, and innovative endpoints. Sixty-two asset-indication examples using these designs were identified across different therapeutic areas. Based on the availability of health technology assessment (HTA) reports and the innovative element’s impact on final HTA outcome, 17 of these identified examples were developed as case studies to highlight the designs’ implications for access. Interviews with eight payer-experts across US, France, Germany, and Japan were conducted to further validate the research, explore scenario analyses, and clarify how circumstances impacted payer acceptability of the studied innovative trial designs.

**Results:**

While oncology has historically spearheaded innovative trial designs, other therapeutic areas are now incorporating innovative elements. Nonetheless, published payer guidance on innovative designs remains limited. We identified seven circumstances impacting payer acceptance of innovative designs: disease prognosis, eligible patient population size, type of treatment, mode of action, availability of treatment alternatives, comparative benefit versus standard of care, and launch sequence. Patient population size had the greatest impact on payer decisions, followed by comparative benefit and type of treatment; this suggests that payers may be inclined to accept innovative trial designs for small populations with high unmet need or therapies with transformative clinical benefits.

**Conclusions:**

Innovative trials trade some scientific validity for increased practicality compared to traditional RCTs. Stakeholders need to align on appropriateness and scientific validity of innovative designs for access decisions, while implementing measures to minimize uncertainties. Collaboration across stakeholders including regulators, payers, and manufacturers is needed to refine innovative methodologies and guide policies to improve the relevance of innovative trials for decision-making.

